# Inequity in clinical research access for service users presenting comorbidity within alcohol treatment settings: findings from a focused ethnographic study

**DOI:** 10.1186/s12939-024-02197-1

**Published:** 2024-05-22

**Authors:** Sofia Hemrage, Stephen Parkin, Nicola J. Kalk, Naina Shah, Paolo Deluca, Colin Drummond

**Affiliations:** 1 Department of Addictions, Institute of Psychiatry, Psychology and Neuroscience, King’s College London, London, UK; 2 Department of Public Health, Environments and Society, London School of Hygiene and Tropical Medicine, London, UK; 3 South London and Maudsley NHS Foundation Trust, London, UK; 4 Institute of Liver Studies, Cheyne Wing (Third Floor), King’s College Hospital, London, UK

**Keywords:** Barriers, Inequity, Access, Research, Clinical care, Service users, Comorbidity, Alcohol use disorder, Alcohol-related liver disease, Focused ethnography

## Abstract

**Background:**

While healthcare policy has fostered implementation strategies to improve inclusion and access of under-served groups to clinical care, systemic and structural elements still disproportionately prevent service users from accessing research opportunities embedded within clinical settings. This contributes to the widening of health inequalities, as the absence of representativeness prevents the applicability and effectiveness of evidence-based interventions in under-served clinical populations. The present study aims to identify the individual (micro), organisational (meso) and structural (macro) barriers to clinical research access in patients with comorbid alcohol use disorder and alcohol-related liver disease.

**Methods:**

A focused ethnography approach was employed to explore the challenges experienced by patients in the access to and implementation of research processes within clinical settings. Data were collected through an iterative-inductive approach, using field notes and patient interview transcripts. The framework method was utilised for data analysis, and themes were identified at the micro, meso and macro levels.

**Results:**

At the micro-level, alcohol-related barriers included encephalopathy and acute withdrawal symptoms. Alcohol-unrelated barriers also shaped the engagement of service users in research. At the meso-level, staff and resource pressures, as well as familiarity with clinical and research facilities were noted as influencing intervention delivery and study retention. At the wider, macro-level, circumstances including the ‘cost of living crisis’ and national industrial action within healthcare settings had an impact on research processes. The findings emphasise a ‘domino effect’ across all levels, demonstrating an interplay between individual, organisational and structural elements influencing access to clinical research.

**Conclusions:**

A combination of individual, organisational and structural barriers, exacerbated by the COVID-19 pandemic, and the socioeconomic landscape in which the study was conducted further contributed to the unequal access of under-served groups to clinical research participation. For patients with comorbid alcohol use disorder and alcohol-related liver disease, limited access to research further contributes towards a gap in effective evidence-based treatment, exacerbating health inequalities in this clinical population.

## Background

An imbalance between evidence-based interventions and their effectiveness in under-served groups presenting comorbidity contributes to widening health inequalities [1]. Patients with comorbid alcohol use disorder (AUD) and alcohol-related liver disease (ARLD) are an under-served clinical group. Under-served groups are those with a lower representation in research in proportion to overall population prevalence and healthcare burden [2]. The absence of representation of these populations in research is rooted in demographic, socioeconomic, health and disease-specific aspects that disproportionately hinder access to clinical resources. Under-served groups are also disproportionately affected by social determinants of health, observed through a social gradient in health status and outcomes across different demographic groups [3]. Population-based studies have reported a disproportionate increase in the prevalence of ARLD during COVID-19 among disadvantaged social groups and ethnic minorities [4, 5]. Socioeconomic barriers and the increases in the cost of living also shape alcohol-attributable health harm [6, 7].

Despite policies to improve access to clinical care and tackle health inequalities, under-served groups still face barriers related to physical disability, co-occurring mental health conditions or socioeconomic status [8, 9]. Previous studies have acknowledged the social and structural challenges in the identification, recruitment and retention of service users from alcohol treatment settings in the clinical research [10]. For example, limited access to telephones (mobile or landline), the latest technology (smartphones) and homelessness often prevent service users from receiving communications, participating in research studies involving digital interventions or being able to store investigational pharmaceuticals in temperature-stable conditions [11, 12]. These barriers bring a lack of representativeness of under-served groups to health research. Consequently, this compromises the generalizability and external validity of the findings, as well as the effectiveness and appropriateness of interventions for certain patient groups [13].

While alcohol consumption patterns determine treatment prognosis, there are also societal and economic aspects that shape alcohol use and ARLD, and can hinder equitable access to health opportunities [14, 15]. When access to healthcare is attainable, this patient group faces additional deterrents to participation in healthcare given their variable pharmacological responses, low treatment compliance and medication overburden [16]. Effective treatment for ARLD remains an unmet need, with limited research being conducted in this clinical population to date from the addictions treatment perspective [17]. This can be explained by limited access to healthcare and research opportunities [18]. Psychosocial interventions present an opportunity to mitigate alcohol-related harm and improve patient outcomes, but evidence of their effectiveness remains scarce [19, 20]. These interventions address substance use through structured, psychological and social approaches such as motivational interviewing, brief interventions and contingency management.

Circumstances resulting from a post-Brexit loss of a European Union workforce, budget cuts to the National Health System (NHS) and concerns over working conditions have resulted in widespread staff shortages in United Kingdom (UK) health settings [21, 22]. In combination with the long-term impact of COVID-19 and the resulting rising demand for medical care, these matters have exerted additional pressure on clinical services [23]. These nationwide issues have been reflected on a day-to-day basis in overbooked clinical lists and extended waiting times for appointments [24].

Embedding research within clinical settings is therefore subject to multiple barriers. Considerable evidence has demonstrated a link between high-risk drinking, the onset of the pandemic and periods of economic crisis [25, 26]. These events have led to a surge in ARLD caseloads, hospitalizations, and mortality [27], placing further strain on the NHS. The above circumstances have been identified as affecting research capacity due to lack of time, insufficient research funding and facilities, as well as excessive paperwork [28, 29]. Additionally, a qualitative study on the impact of the pandemic on clinical research has noted that changes to collaboration and the loss of workforce following COVID-19 have disrupted the conduct of studies within clinical and academic settings [30].

### Role of qualitative research in understanding inequity in clinical research access

Qualitative research can facilitate the understanding of the social phenomena occurring within settings such as health systems. It provides an interpretative insight into the complex interplay between elements within a system, and how global, national, and local health policies are implemented and experienced in clinical care. Correspondingly, the value of qualitative methods for the study of equity in health lies in their ability to capture the intersection between cultural, structural, and socioeconomic elements and health outcomes [31, 32].

Ethnographic research is proven to be optimal for engaging with under-served populations and settings, substantiating research on health inequalities [33]. Ethnography is a qualitative methodology that allows the understanding of interactions, organisations and verbal and visual conduct through immersion within the context of interest [34]. This requires long-term fieldwork, which is not always viable due to time and resource constraints. An adaptation of ethnographic work to fast-paced settings, such as hospital wards, is focused ethnography [35]. Focused ethnography is a pragmatic and feasible qualitative methodology, whose limited timeframe and context-specific nature enable an understanding of specific social mechanisms within the context of clinical care and applied research [36]. The use of focused ethnography as a qualitative mode of inquiry has become increasingly well-established as a method to study social interactions within healthcare settings [37, 38].

### Current study

The current study draws on qualitative data from a prospective, individually randomized pilot trial involving patients with comorbid AUD and ARLD (NCT06183710) [39]. The pilot study aimed to assess the feasibility of contingency management to improve treatment adherence in this patient group. Contingency management is a psychosocial intervention in which gradual, increasing reinforcers are provided upon evidence of a certain behaviour, such as medication compliance or treatment adherence [40]. Following hospital admission, participants were randomised to the control group, in which they received outpatient integrated care only, or to the intervention group, in which patients received the contingency management intervention in addition to integrated care. Integrated care incorporates both liver and addiction services within the same outpatient clinical setting, having an essential role in the treatment of ARLD. The integrated care follow-up was delivered for 3 months, with follow-up points at the end of the intervention and 3 months later.

Despite the design of the study integrating feedback from service users to minimise attrition, the research team encountered obstacles in the research concerning access, participation, and participant retention. In the present article, the authors employ a focused ethnography approach to explore and systematically dissect these challenges and translate the findings into applied research.

An iterative-inductive approach to focused ethnography is applied in this paper [41]. While this model has been established, there is a scarcity of studies employing this model in health and medical research, filling a methodological gap in qualitative research. The use of an iterative-inductive approach to ethnographic analysis enhances data validity and trustworthiness by enabling triangulation across different sources [42].

To the authors’ knowledge, this is the first study to apply focused ethnography to document both the barriers to engaging under-served clinical populations in research and the challenges of embedding research within clinical care [43, 44]. The overall aim of this research is to explore the impact of a conjunction of socioeconomic and environmental barriers and potential facilitators on conducting research within clinical settings, and how these may hinder health equity. Resorting to a social systems, multi-level framework through the initial identification of individual (micro), institutional (meso), and structural (macro) barriers to conducting alcohol research within clinical settings, the authors explore the underlying causes of inequity in a specific, real-world clinical care setting), among a population that has been widely excluded from research. Additional aims of this qualitative inquiry include the understanding of the meaning of research participation and circumstances underlying the exclusion of patients with AUD and ARLD from research. In addressing these aims, the authors also intend to reflect on the contribution of unequal access to clinical research to health inequalities, from a patient-centred perspective.

## Methods

### Methodology: focused ethnography

An increasing number of health research studies have adopted focused ethnography as a pragmatic approach to conventional ethnography [45, 46]. Focused ethnography is characterised by short-term field visits, frequently conducted in intervals of focused exploration. Data availability is ensured through triangulation of information through multiple data collection methods, such as field notes and in-depth interviewing.

### Iterative-inductive model of focused ethnography

The iterative-inductive approach recognises a mutual exchange between theory and observation [47]. The model acknowledges the strengths of inductivism while enabling a simultaneous deductive approach to the phenomena [48, 49]. In focused ethnography, an iterative-inductive approach strengthens data collection allowing a comprehensive insight into the research inquiry through deductive and inductive mechanisms [50]. As such, it is conveniently aligned with the time-bounded nature of focused ethnography, optimising the availability, quality, and validity of the data.

### Design

Data for this study were generated using a focused ethnography approach as applied in previous ethnographic research conducted on healthcare settings [38]. Between January and December 2023, SH obtained a detailed insider view by incorporating an observant participation role within a hospital setting [51–53]. By working as an embedded researcher in the clinical setting, the observant participation role carried by the researcher prioritised observation over participation, relying on familiarity with the setting as opposed to full immersion in an unfamiliar context [54]. Themes that emerged from the data were analysed thematically.

The study follows the Standards for reporting qualitative research (SRQR) recommendations [55] (Appendix 1).

### Setting

This study took place across various inpatient wards within a large south London teaching NHS Trust hospital, part of a wider academic health science network of centres across London. As part of the NHS, the trust provides publicly funded healthcare to legal UK residents, serving a population of approximately 1,008,700 south London residents [56]. The hospital’s bed capacity is over 1300.

### Sample

Consecutive sampling was used to recruit 30 participants for the pilot feasibility study. The target population were adult individuals with a clinical presentation of comorbid AUD and ARLD, admitted to the hospital. Identification and referral of potential participants was facilitated by Consultant Addictions Psychiatrist (NJK) and Consultant Hepatologist (NHS) within the Alcohol Care Team, who discussed the study with patients. A participant information sheet was provided, and prospective patients were given at least 24 hours to consider the study. The research team (SH, NJK, NS) would then approach prospective participants again and seek written consent. Table 1 describes the eligibility criteria for the pilot study:

**Table 1:** Eligibility criteria for the pilot feasibility trial of contingency management to incentivise treatment adherence in ARLD

**Inclusion criteria**
a) 18 years old or above.
b) Able to communicate in English independently.
c) Formal diagnosis of ARLD in line with ICD-10 K70 codes (fatty liver, hepatitis, fibrosis, sclerosis, cirrhosis, hepatic failure, unspecified liver disease) upon discharge following acute liver disease episode.
d) Formal diagnosis of AUD in line with ICD-10 F10.2 codes.
e) Attending South London liver services (King’s Health Partners).
f) Able and willing to provide informed consent.
g) Able and willing to participate in the study.
h) Owning a mobile phone.
**Exclusion criteria**
a) Less than 18 years old.
b) Current dependence on other substances other than alcohol, tobacco, or cannabis
c) Not able to communicate in English independently.
d) Being pregnant.

In the interests of clarity, the authors reiterate that the sample described above pertains to the pilot study, and hence it is not specific to the ethnographic component of the present study.

### Data collection and analysis

A combined approach to data collection was taken to allow methodological and data source triangulation. Field notes and interview data were collected by a female researcher (SH) who had conducted preliminary shadow work within the clinical setting. The researcher received the relevant training before starting the pilot trial. Through an observant participation role, the continued and collaborative partnership established familiarity with the healthcare professionals and with the clinical environment. This enhanced the reflexivity of the researcher the present ethnographic investigation. Previous engagement with the setting also allowed patients and staff to become acquainted with the presence of the researcher, minimizing potential sources of bias stemming from lack of candour (Hawthorne effect) [57, 58].

Field notes, consistent with a focused ethnography methodology, were written in a retrospective reflective journal. These included descriptions of behaviour, interactions and relevant events across different settings, subjects, and time points. The field notes substantiating the ethnographic component of the present investigation were written at the end of each day during the period in which recruitment for the pilot study took place (January to December 2023). During this period, the researcher accompanied clinicians during clinical reviews, ward rounds and handover meetings.

Following enrolment in the study, an in-depth, semi-structured interview was carried out by the female researcher (SH). The interviews aimed to understand barriers and enablers to treatment adherence, as well as participants’ attitudes towards contingency management. Interviews were recorded using an encrypted device and transcribed verbatim by a professional transcription service. In addition to the methodological and data source triangulation above mentioned, interview data were triangulated via researcher triangulation. This involved comparison of commonalities and differences across emerging themes and subsequent discussion of these within the research team. Interview excerpts included to support the field notes are solely from patient participants in agreement with the patient-centred focus of this article.

Data from all sources were managed and organised with the qualitative data software program NVivo 11. Data analysis involved the Framework method in which the first author followed the steps advocated by Ritchie and Spencer [59]. Namely, the first author followed the recommended processes of data familiarization, framework identification, indexing and charting data, and interpretation [60].

The analytic frame adopted in this study was multilevel analysis (MLA), a social systems framework which has been well-established approach in healthcare qualitative research [61–63]. MLA allowed the identification, integration, and conceptualisation of the interplay between the micro (service users), meso (clinical setting), and macro (environment) processes. The proposed levels of analysis are defined as follows:

- *Micro-level determinants: *the individual level comprising of the interaction between service users and research. This includes all aspects occurring at the individual level and shaping service user’s involvement and participation in research (clinical presentations, psychological, social and financial barriers).
- *Meso-level determinants:* the interface between the institution and research processes. For the present study, the institution is defined as the trust in which the pilot study was conducted, its personnel and internal policies.
- *Macro-level determinants:* refer to the structural forces, beyond the individual and institutional control, but may still influence research processes and care delivery.

### Ethical considerations

The pilot study has been reviewed by King’s College London and King’s College Hospital following the research ethical standards in place. A favourable ethical opinion was granted by Camden and Kings Cross NHS Research Ethics Committee (reference 22/LO/0744).

## Results

**Fig. 1:**
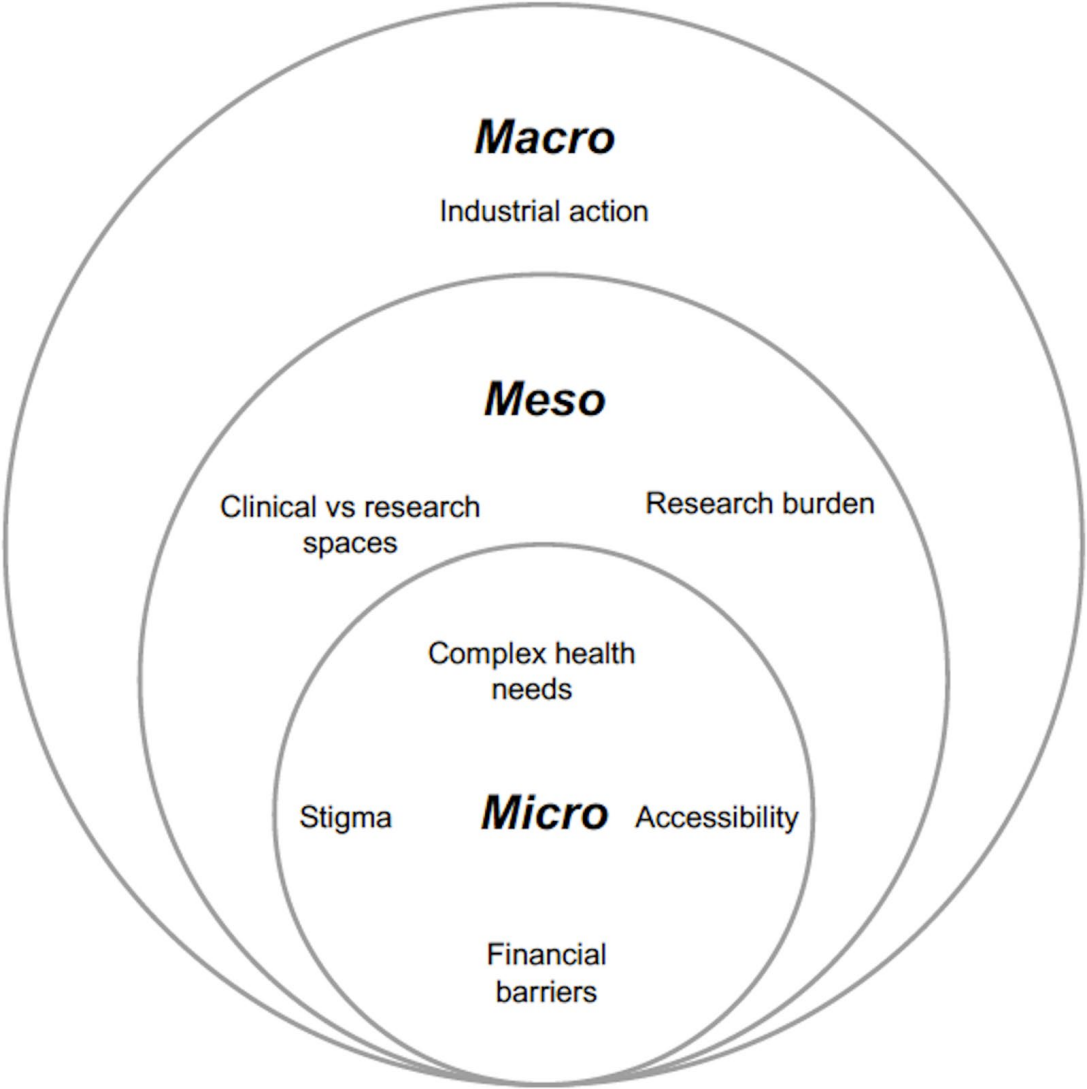
Identified micro, meso and macro-level barriers to clinical research access and participation in service users with AUD and ARLD

Through observant participation, barriers at the micro, meso, and macro levels were encountered, influencing both research processes (sampling, recruitment, intervention delivery, data collection) and clinical care. This is illustrated in Fig. 1:

### Micro-level determinants: the interface between the service user and research participation

#### Complex health needs

An observation from the fieldwork was that although alcohol-related barriers to research were encountered, alcohol-unrelated barriers presented greater challenges to patient engagement, as discussed in the next section.

Alcohol-related barriers were linked to alcohol withdrawal delirium, Wernicke’s or hepatic encephalopathy, and alcohol-related brain damage, which rendered patients unable to participate in research. At the micro-level, alcohol withdrawal delirium and Wernicke’s encephalopathy presented temporary barriers to engagement as the research team refrained from engaging with patients until such symptoms improved. As a result of the non-linear trajectory and individual variability of recovery, unpredictability also shaped the flow of the research. This may be noted in the following field note relating to the stages of approach and consent:

[Fieldnote entry, P8, male, pre-enrolment]: “(…) He is currently experiencing withdrawal symptoms and given his previous admissions, was advised to wait further until his condition stabilises. His symptoms (alcohol withdrawal delirium) had escalated overnight and a DoLS (Deprivation of Liberty Safeguards) was put in place.”

Alcohol use can worsen pre-existing health conditions, by inducing or contributing towards additional comorbidities in patients with comorbid AUD and ARLD. Alcohol-unrelated barriers included age-related dementia. This would prevent participation in research during the stages of approach and obtaining informed consent, as participants often presented difficulties in recalling study information and previous visits research team. These patients were also precluded from participating in research as the ability to provide informed consent is an inclusion criterion for the pilot study.

For many patients, the primary medical cause underlying their admission was not exclusively alcohol-related and included presentations such as fractures or diabetic neuropathy. Nevertheless, their comorbid AUD and ARLD diagnosis led to a referral to the Alcohol Care Team, and subsequently to the pilot study. The complexity and multifaceted clinical presentation of these potential research participants meant that patients were undergoing routine medical examinations or procedures beyond alcohol treatment. Thus, it would often be the case that patients were not physically present in the ward, and therefore temporarily unavailable to be approached and participate in the study:

[Fieldnote entry, P14, female, P15, male, pre-enrolment] "Went to the ward and attempted to approach the patient this morning but did not find patient as she had been taken for an ultrasound (…) when trying to see the patient alongside the clinical team this morning, she was not by her bed - the nurse said she had been taken for a CT scan (…) they were about to be taken into the theatre.”

As a repercussion of the burden of coexisting illness, participants often placed AUD and ARLD as secondary, and thus afforded less clinical priority to their management. When approached, patients frequently found themselves overloaded with medical treatment and information related to their primary medical presentations. Recovery from these is therefore an immediate therapeutic priority, overtaking alcohol treatment and research participation. These situations of overburden and reduced priority may be noted in the following fieldnotes:

[Fieldnote entry, P11, male, baseline data collection]: “The patient refused his copy of the consent form, saying "I don't want any more papers, I already have enough” and showed me his medical forms and prescriptions.”

[Baseline interview excerpt, P3, male]: “I think I’m more concerned with my kidneys.”

An additional alcohol-unrelated barrier emerging at the micro-level was communication, reflecting on the patient’s ability to engage independently, and subsequent capacity to understand research documentation and verbal communication (such as providing informed consent). This communication barrier often stemmed from low literacy rates and participants’ preferred language, preventing patients from understanding research-related documents independently:

[Fieldnote entry, approached male patient in hospital ward]: “(…) he expressed that although he understood the study he was unable to read if provided with the participant information sheet and consent form”

#### Accessibility

The multimorbidity presented by some patients created a hurdle to access the care provided as part of the research intervention. Despite being motivated to participate in the pilot trial and engage with the integrated liver clinic, some patients anticipated that accessibility would be a constraint to attending hospital visits, even if patient transport systems were available:

[Fieldnote entry, P6, male, informed consent stage]: “The patient had a few queries related to the study, especially related to accessibility. Despite being highly motivated, he was diabetic and had recently undergone a procedure on his foot. I suggested the hospital’s patient transport system and provided the patient with their contact details.”

Some patients also anticipated that taking public transportation to attend clinical appointments would be distressing and anxiety-inducing.

#### Financial barriers

Findings obtained while conducting focused ethnography within the pilot study are coherent with wider literature on the inverse gradient between alcohol-related harm and socioeconomic status. Financial hardship becomes heightened with the sharp increase in the cost of living during 2022 and 2023, whose effects were widely reported at the time of fieldwork [64]. Under the circumstances, research participation was premised upon the ability of participants to attend the integrated liver care clinic appointments; concerns anticipated by patients were transportation costs to attend the hospital, as well as a reluctance to be absent from work, as illustrated below:

[Fieldnote extract, P17, female, pre-enrolment]: “The patient worried that she could only participate if appointments were on specific days of the week, as she was working and could not miss work otherwise would not get paid (…)”.

[Baseline interview excerpt, P2, male]: “Mobility is a physical bar to attending appointments. If I can’t get hospital transport, I just can’t go because my finances prohibit me from taking taxis to some places. Particularly a taxi to the hospital would be £40 or something, return £60, obviously I can’t afford that.”

Homelessness and digital exclusion were also barriers to research participation, preventing patients from receiving hospital communications such as information with appointment details. In situations where service users did not own a mobile phone nor had access to e-mail, communication would be attempted via post or through assigned key workers. As observed, community alcohol services were also unable to contact service users as they did not have mobile, landline, or e-mail access. As a result, these participants were unable to fully engage in the research study.

#### Stigma

At the micro-level, stigma-related issues surrounding AUD emerged and appeared to influence research processes. This was observed at all stages of fieldwork - from the initial approach to engagement with research follow-up visits. Some patients, despite previously attending alcohol treatment services, were concerned about the presence of other patients:

[Fieldnote entry, P9, female, pre-enrolment]: "She requested not to mention the word “alcohol” as she did not want other patients in the ward to know she was alcohol dependent; we agreed to meet in one of the clinical examination rooms of the outpatient department the following week, to preserve her privacy and allow a comfortable space for the patient to talk."

Research participation and engagement also raised privacy concerns for some patients. For example, since their relatives and social circle were not aware of their alcohol-related diagnoses, some patients were ambivalent about participating in research, as receiving integrated liver care follow-up could alert their friends and families to conditions previously not disclosed:

[Fieldnote entry, P9, female, pre-enrolment]: “The patient worried that her mother would find out she had relapsed if she continued to be involved in the research."

Worrying about stigma led to patients withdrawing their consent to participate during the pilot study as they were apprehensive about receiving research and hospital-related communication at their home addresses.

### Meso level determinants: interface between clinical care and research

During this study, the clinical team were impacted by strikes, staff sickness, and the implementation of a new electronic health record system, which meant that they often prioritised clinical work. A surge in caseloads at the integrated clinic was observed between the time the study was planned compared to when it was conducted. Further effects have been visible while implementing the research within clinical care; for the pilot study, this resulted in a time lag in providing clinical care follow-up appointments to the enrolled patients, as well as a lack of availability of facilities to conduct both research data collection and follow-ups. This is partly related to the cancellation of all clinics during strike periods and the implementation of the new record system.

### Role of place: clinical vs research physical spaces

To mitigate full clinic lists and the lack of availability of facilities to deliver integrated liver care as part of the research study, the research team set up an ad hoc research clinic. The clinical research space had a different location to the outpatient liver clinic, with which patients were familiar and accustomed. The research clinic allowed for a more inclusive, patient-centred approach, given that patients had the option of choosing a more convenient timeslot to attend. Retention rates of participants at the clinical research space were lower compared to the outpatient liver clinic, hinting at ambivalence towards clinical care delivered as part of the research or belief that research participation would preclude clinical care, as described below:

[Fieldnote entry, P14, female, study follow-up]: “A few minutes before her visit, the patient called the research team and expressed that she would not be able to attend and would prefer to be seen at the outpatient department.”

### Research burden

The heterogeneity of clinical presentations, as well as its interdisciplinary management, involving both hepatology and addiction care, compels research to approach ARLD from different perspectives. At the time of the pilot study, two other competing liver and addictions focused RCTs were being conducted within the same clinical setting. Despite being associated with perceived benefits, research participation can be obtrusive and a source of burden for service users, as noted in the findings. While conducting the pilot study, this was particularly the case at the stages of approach and pre-enrolment of potential participants. Frequently, this culminated in the inadvertent overlapping of research processes from different trials, as evidenced in the following fieldnote:

[Fieldnote entry, P2 and P16, both male, baseline data collection]: “Went to the patient’s room, patient was being seen for a blood research study. Waited outside until the tests were over. (…) The interview was slightly disrupted by a device beep and a research nurse coming in to take blood tests for another research study; came back a few hours later, patient was having his blood taken; waited outside as another research nurse was collecting the patient's blood as part of another liver trial.”

[Baseline interview excerpt, P2, male]: (…) as you’ve noticed there’s always been a queue of people waiting to come in and see me.”

Research overburden also appeared to lower overall recruitment rates to the study given the similar eligibility criteria targeting a specific group of patients within a specific hospital setting. For research teams, who are required to meet recruitment targets on behalf of sponsors, this adds a competing, territorial element to patient recruitment.

From the meso-level perspective, the varied studies in place amounted to an increasing research burden on the clinical population of interest, and blurring the boundary between clinical care and research:

[Fieldnote entry, P16, male, baseline data collection]: “Asked patient if he was available to have a chat about the study, to which patient said yes, but was confused about the study which I was referring to as "there's so many. (…) Earlier today they came to get blood and I thought it was for my medical care, but it was just research”. The patient seemed overwhelmed and burdened with research documents - I could see the various information sheets and questionnaires by his bedside.”

It would often be the case that when approaching a patient, the researcher would be asked to ‘come back on another occasion’ as they had already participated in research-related processes that day.

### Macro-level determinants: the interface between environment and research

#### Industrial action

The socioeconomic landscape in which the research was conducted also influenced its implementation and management. Stemming from structural forces, the combination of the cost-of-living crisis, rising inflation and a demand for better working conditions prompted industrial action across the healthcare, education, and transportation sectors [65].

Industrial action within the healthcare sector included strikes among doctors, nurses, ambulance workers and NHS Trust staff. These had the most tangible effects at the macro-level, resulting in disruptions within clinical care:

[Baseline interview excerpt, P3, male]: There’s a strike on at the moment which doesn’t really help (...) It’s very difficult to tell how things have changed, because you’re just a patient. To turn round say ‘you need to do this, you need to do that’ you can’t really say that because you don’t know the situation that some of those nurses are in.”

For the pilot study, this implied that staff involved in the research were not always available due to staff cover and the need to prioritise clinical care for safety reasons. All outpatient clinics were cancelled on strike days, which led to the re-scheduling of the integrated liver care follow-ups in the ad hoc research clinic.

Indirectly, industrial action in the transportation and education sectors meant that both patients and clinical staff had difficulties accessing the hospital for clinical follow-up appointments or had to fulfil childcare responsibilities. Industrial action was therefore a phenomenon stemming from structural reasons which had an impact on the internal dynamics within the hospital, and consequently, on the pilot study.

## Discussion

The current study identified the existing barriers to engaging service users presenting AUD and ARLD in a pilot study conducted within a clinical setting. By considering the micro, meso and macro levels of analysis, this qualitative exploration of the challenges to engage service users in clinical research recognizes the versatility of applying an iterative-inductive model of focused ethnography to research conducted in fast-paced, dynamic clinical environments. The findings also provide a deeper understanding of the impact of COVID-19 and the UK’s socioeconomic landscape on research, clinical care, and health inequalities from a patients’ perspective.

An observation to surface from the data was the contrast between alcohol-related and unrelated barriers to participation in research at the micro-level. While confusion related to acute withdrawal delirium, Wernicke’s or hepatic encephalopathy may serve as transient obstacles to research participation, alcohol-unrelated issues such as coexisting illness, chronic cognitive impairment and communication barriers establish more pervasive barriers to research participation. For research, these barriers have a tangible effect at the stages of approaching and obtaining informed consent, which requires information retention and decision-making capacity. This is consistent with a wide range of previous research, pointing that acute withdrawal, comorbid health conditions, chronic brain changes from long-term substance use and limited educational attainment delineate understanding and retention of consent information, as well as data collection [66–69].

Participants’ ability to maintain capacity during the study is also subject to variation. Hepatic encephalopathy, a confusional state related to the translocation of bacterial toxins from the gut and their inadequate filtering by the liver, is a complication of advanced ARLD and was a particular challenge because of its fluctuating states and could occur at any point during the study. At the initial stages of the research, this affects the understanding of study-related information, decision-making and subsequent capacity to provide informed consent. A proposed strategy to address this challenge is an ongoing evaluation of cognitive capacity, with the development of standardised tools to evaluate and grade hepatic encephalopathy during a patient’s clinical trajectory [70].

Participation in research embedded within healthcare can also be limited by coexisting economic and societal barriers. The centralisation of health services and resources presupposes physical mobility as a requisite for access. A strong correlation between a lack of access to adequate mobility and a lack of access to opportunities, health-enabling resources, and services has been consistently reported in the literature [71]. This link occurs both as a cause and consequence of social exclusion, materializing in health disparities across different population groups [72]. As a social determinant of health, social exclusion is a driving factor for health inequalities [73, 74]. Through observant participation in this study, such circumstances were made evident in terms of how accessibility, transportation and affordability underlined research access and participation. While a universal, free, publicly funded healthcare system and initiatives such as integrated care and patient transport seek to overcome these challenges, there is still a strong association between social exclusion, accessibility and unequal engagement with health services, and respective embedded research [75]. A similar study found that transportation, inability to take time off from work, schedule conflicts, lack of childcare and provider beliefs as barriers compromising participation in research [76]. For health policy and clinical practice, the translation of the findings obtained from the current focused ethnography coupled with those of previous research suggests that lack of participation in health research perpetuates disparities in health service use and research access.

While virtual mobility can provide an alternative to physical mobility, another factor compounding social exclusion is the unequal access and capacity to use information and communication technologies [77]. As observed in this investigation, digital exclusion reinforced unequal access to resources, including research and medical care [78]. Specifically, lack of mobile or smartphone ownership or access to e-mail amongst patients with current experience of homelessness impacted levels of engagement with both the pilot study and clinical care, as well as engagement with community drug and alcohol treatment teams. Future research could allocate funding for the provision of communication technologies. A qualitative study has shown that this improved research participation among under-served groups [79]. Although noted in this study, these findings may not be generalised to a wider spectrum of service users. While mobile ownership in vulnerable groups is limited, several cross-sectional studies noted utilization rates of above 80% among individuals attending substance use treatment [80, 81].

The culmination of different social attitudes and negative beliefs towards AUD in a common, shared environment delineated engagement and participation in the pilot study. It has been widely established that stigma is a barrier to seeking treatment for AUD, contributing to a gap between those who are affected and those who are in treatment [82–84]. While policy-oriented strategies have focused on addressing structural stigma within services and organisations, internalized negative beliefs towards oneself (self-stigma), overt and anticipated fear of discrimination or social rejection (public stigma) still serve as impediments to engagement and adherence [85]. The effect of stigma in research-related processes, such as recruitment and intervention delivery, was evident through observation and interviewing. These findings uphold the need to evaluate the role of reflexive, internalized emotions on self-stigma among vulnerable substance use groups and the involvement of social networks in the management of AUD and ARLD [86, 87].

The above barriers have been exacerbated by wider national elements such as the impact of COVID-19 and economic recession. Conducting a pilot study in an overstretched system further revealed the effect of NHS staff and resource pressures on research-related procedures. Where healthcare services face constraints to meet a rising clinical demand, research within care will also be affected. The findings raise concerns regarding the future of health services and implementation research, in agreement with a reported 44% decline in patient access to healthcare research [88]. In real-world settings, this is detrimental to patient groups with limited access to evidence-based, innovative treatment pathways.

The study also emphasized the interplay and influences between micro, meso and macro barriers. A clear-cut example of this was the ‘domino effect’ prompted by the cost-of-living crisis on the interfaces between service users, clinical care and environment and research. At the micro-level, financial hardship and living costs contributed to further exclusion from research and care, hindering service users from accessing beneficial clinical research opportunities. Financial pressures have also implied healthcare budget cuts and subsequent staff and resource shortages at the meso-level. At the wider, macro-level, the increasing cost of living has prompted industrial action across different sectors. Conversely, the cost of living has also impacted service users’ ability to access health resources, whether by increasing transportation costs or experiencing homelessness. This demonstrates the transversality of a financial factor across different levels, and how the dynamic interplay across these can shape equity in access to health-enabling resources. Therefore, while the identified barriers can be used to improve equity in health, these should also be mapped based on relationality and interconnectedness [89].

An additional example of the interaction between layers pertains to the research burden observed at the institutional (meso-level). Although striving to conduct research to improve health outcomes at the individual (micro-level), the competing environment of health research often results in compartmentalization and lack of collaboration across research teams [90]. These may be a symptom of wider, macro-level structural elements within the neo-liberal context of competition for research grants, established across conceptual and empirical literature [91–93].

One of the key strengths of this study is the suitability of its methodological approach and its scope to inform equity-oriented, health services research. This investigation sits on a methodological spectrum ranging from exploration to theory building, fed by a circular, reflexive approach. Observant participation, inherent to a focused ethnography approach, allowed a first-hand, comprehensive understanding of the challenges that health research is subject to when embedded within clinical settings as well as the wider, structural drivers of unequal access to health-enabling opportunities. The urgency of these barriers becomes paramount within a post-pandemic, socioeconomic volatile context, tied to the widening of health gaps across population groups. Yet, as this study was conducted in a large hospital allied to a university in a major city, extrapolation of the findings may not be generalisable to other settings. Further work is therefore needed to ascertain the variability of these barriers with differences in organisation and practice across clinical settings, as well as within remote, socioeconomically deprived areas. Notwithstanding, the overall work provides valuable insights into the socioeconomic, systemic, and structural determinants that persist and prevent under-served groups from accessing clinical research opportunities, and how this inequitable access may perpetuate health disparities.

## Conclusions

Overall, the findings of this study allow a multifaceted understanding of the determinants that shape the engagement of under-served clinical populations with complex health needs in research, and how the surrounding socioeconomic UK landscape delineated the hybridization of research within clinical care. At the micro-level, aspects such as complex clinical presentations, accessibility, financial barriers, and stigma were found to hinder access and participation in research. At the meso-level, while the research burden compromised participants’ ability to engage, the findings suggest that familiarity with the space in which research is being conducted may improve participation. At the structural, and macro-level, industrial action was noted to impact research access and respective processes. The findings also highlight that the identified barriers do not exist in isolation, but rather interact across different levels. An example of such interaction is the financial challenges encountered at the individual, institutional and structural dimensions.

Using a focused ethnography approach, a myriad of individual, organisational and structural aspects shaping access and engagement in research were observed. The effect of these determinants was augmented following the COVID-19 pandemic and the socioeconomic landscape in which the study was conducted. Consequently, they condition the access of under-served groups to clinical research. For patients with AUD and ARLD, limited access further contributes towards a gap in effective evidence-based treatment, reinforcing health inequalities in this clinical population.

## Data Availability

No datasets were generated or analysed during the current study.

## Appendix 1: Standards for Reporting Qualitative Research (SRQR) checklist [55]

**Table:**

	**Page #**
**Title and abstract**	
**Title** - Concise description of the nature and topic of the study Identifying the study as qualitative or indicating the approach (e.g., ethnography, grounded theory) or data collection methods (e.g., interview, focus group) is recommended	1
**Abstract** - Summary of key elements of the study using the abstract format of the intended publication; typically includes background, purpose, methods, results, and conclusions	2
**Introduction**	
**Problem formulation** - Description and significance of the problem/phenomenon studied; review of relevant theory and empirical work; problem statement	3
**Purpose or research question** - Purpose of the study and specific objectives or questions	7
**Methods**	
**Qualitative approach and research paradigm** - Qualitative approach (e.g., ethnography, grounded theory, case study, phenomenology, narrative research) and guiding theory if appropriate; identifying the research paradigm (e.g.,postpositivist, constructivist/interpretivist) is also recommended; rationale	8
**Researcher characteristics and reflexivity** - Researchers’ characteristics that may influence the research, including personal attributes, qualifications/experience, relationship with participants, assumptions, and/or presuppositions; potential or actual interaction between researchers’ characteristics and the research questions, approach, methods, results, and/or transferability	11
**Context** - Setting/site and salient contextual factors; rationale	9
**Sampling strategy** - How and why research participants, documents, or events were selected; criteria for deciding when no further sampling was necessary (e.g., sampling saturation); rationale	9
**Ethical issues pertaining to human subjects **- Documentation of approval by an appropriate ethics review board and participant consent, or explanation for lack thereof; other confidentiality and data security issues	13
**Data collection methods** - Types of data collected; details of data collection procedures including (as appropriate) start and stop dates of data collection and analysis, iterative process, triangulation of sources/methods, and modification of procedures in response to evolving study findings; rationale	11
**Data collection instruments and technologies** - Description of instruments (e.g.,interview guides, questionnaires) and devices (e.g., audio recorders) used for data collection; if/how the instrument(s) changed over the course of the study	11
**Units of study** - Number and relevant characteristics of participants, documents, or events included in the study; level of participation (could be reported in results)	9
**Data processing** - Methods for processing data prior to and during analysis, including transcription, data entry, data management and security, verification of data integrity, data coding, and anonymization/de-identification of excerpts	10
**Data analysis** - Process by which inferences, themes, etc., were identified and developed, including the researchers involved in data analysis; usually references a specific paradigm or approach; rationale	12
**Techniques to enhance trustworthiness** - Techniques to enhance trustworthiness and credibility of data analysis (e.g., member checking, audit trail, triangulation); rationale	12
**Results/findings**	
**Synthesis and interpretation** - Main findings (e.g., interpretations, inferences, and themes); might include development of a theory or model, or integration with prior research or theory	13
**Links to empirical data** - Evidence (e.g., quotes, field notes, text excerpts, photographs) to substantiate analytic findings	13-22
**Discussion**	
**Integration with prior work, implications, transferability, and contribution(s) to the field -** Short summary of main findings; explanation of how findings and conclusions connect to, support, elaborate on, or challenge conclusions of earlier scholarship; discussion of scope of application/generalizability; identification of unique contribution(s) to scholarship in a discipline or field	22
**Limitations** - Trustworthiness and limitations of findings	26
**Other**	
**Conflicts of interest **- Potential sources of influence or perceived influence on study conduct and conclusions; how these were managed	30
**Funding **- Sources of funding and other support; role of funders in data collection, interpretation, and reporting	29

## Abbreviations

ARLD: Alcohol-related liver disease
AUD: Alcohol use disorder
NHS: National Health System
UK: United Kingdom
